# The Erythrocyte Sedimentation Rate as a Novel Prognostic Marker in Canine Inflammatory Diseases

**DOI:** 10.3390/ani16010040

**Published:** 2025-12-23

**Authors:** Jae-Beom Joo, Keon Kim, Woong-Bin Ro, Chang-Min Lee

**Affiliations:** 1 Department of Veterinary Internal Medicine, College of Veterinary Medicine and BK21 FOUR Program, Chonnam National University, Gwangju 61186, Republic of Korea; 2 Department of Veterinary Clinical Sciences, College of Veterinary Medicine, The North Carolina State University, Raleigh, NC 27607, USA

**Keywords:** dog, erythrocyte sedimentation rate, systemic inflammation, prognosis

## Abstract

Inflammatory diseases are common in companion dogs. Although white blood cell (WBC) count and C-reactive protein (CRP) are routinely used to assess systemic inflammation, their individual prognostic value remains limited. The erythrocyte sedimentation rate (ESR), reflecting red blood cell aggregation driven by plasma proteins, has long been applied in human medicine as both an inflammatory and prognostic marker. This study evaluated the prognostic utility of ESR in dogs, including clinically healthy and diseased populations. Associations between ESR, other inflammatory markers, and age were examined, and its role as an independent predictor of mortality and optimal clinical cut-off were determined. A subgroup of dogs meeting the systemic inflammatory response syndrome (SIRS) criteria were also analyzed to assess the latter’s usefulness in acute settings. A total of 350 dogs were enrolled: 241 diseased and 109 healthy. ESR was measured using an automated analyzer. Receiver operating characteristic (ROC) curve analysis, expressed as the area under the curve (AUC), evaluated diagnostic accuracy, followed by Kaplan–Meier survival and Cox proportional hazard analyses. ESR values were significantly higher in the disease group than in the healthy group (*p* < 0.0001). ESR showed fair to good prognostic accuracy for mortality in both the overall cohort (AUC = 0.776 [95% CI: 0.709–0.842]) and the SIRS subgroup (AUC = 0.846 [95% CI: 0.747–0.946]). An ESR cut-off of 18 mm/h was associated with mortality in SIRS dogs with 87.5% specificity. In the multivariate analysis, ESR showed an independent association with mortality (hazard ratio 1.013 [95% CI: 1.004–1.022], *p* = 0.004). These findings support ESR as a practical and independent prognostic marker for risk stratification in dogs with systemic inflammation.

## Simple Summary

Inflammatory diseases are frequently encountered in dogs, yet standard markers like white blood cell counts and C-reactive protein have limitations in predicting patient survival. The erythrocyte sedimentation rate (ESR) is a well-established test in human medicine for assessing inflammation and prognosis; however, its prognostic value in veterinary medicine remains underexplored. This study evaluated the potential of ESR as a prognostic biomarker in 350 dogs, including both healthy individuals and dogs with various underlying diseases, including those with systemic inflammation. The results showed that ESR levels were significantly higher in diseased dogs compared to healthy controls. Notably, elevated ESR levels were associated with higher mortality rates, particularly in dogs with systemic inflammatory response syndrome. Specifically, an ESR value exceeding 18 mm/h was a strong predictor of poor outcomes in these critically ill patients. These findings suggest that ESR serve as a practical and valuable tool for risk stratification, offering veterinarians a useful indicator for predicting prognosis in dogs with systemic inflammation.

## 1. Introduction

Inflammatory diseases are among the most frequently encountered clinical conditions in companion dogs. Various hematologic and biochemical markers, including white blood cell (WBC) count and C-reactive protein (CRP), are routinely used as indicators to quantify inflammatory activity [1]. However, increases in these individual inflammatory markers do not necessarily indicate poor prognosis. Consequently, recent studies have focused on developing various combinations based on these parameters or identifying novel biomarkers to evaluate their prognostic utility in veterinary medicine [2].

Among inflammatory markers, the erythrocyte sedimentation rate (ESR) is a well-established parameter that reflects red blood cell aggregation influenced by plasma proteins such as fibrinogen [3]. More specifically, the ESR represents a hematological measurement of the vertical settling rate of erythrocytes in anticoagulated blood and is directly modulated by acute-phase proteins—including fibrinogen and immunoglobulins—which reduce the zeta potential of red blood cells and thereby accelerate sedimentation [3]. It has long been used as a representative indicator of systemic inflammation in human medicine [3,4]. In human medicine, the ESR is widely recognized as a nonspecific but integrative marker that reflects the cumulative burden of inflammation and correlates with disease activity and prognosis across various disorders, including infections, autoimmune diseases, and malignancies [4,5,6]. Recent veterinary studies have begun to explore its diagnostic utility in inflammatory diseases [3,4,6], yet reports remain limited, and its prognostic value has not been systematically evaluated.

Within the spectrum of inflammatory conditions, severe inflammatory states—particularly systemic inflammatory response syndrome (SIRS)—are clinically important indicators of disease severity and progression [7,8]. SIRS involves dysregulated inflammation that may progress to multi-organ dysfunction [7,8,9,10], and in these systemic inflammatory states or multiple-organ dysfunction syndrome, the ESR has been identified as an important prognostic marker that reflects the severity of inflammation and predicts adverse outcomes and mortality [4,5,6,11,12,13]. These findings suggest that the ESR may serve not only as a marker of inflammation but also as a valuable prognostic indicator in specific disease contexts within veterinary medicine.

This study investigates the clinical and prognostic significance of the ESR as an inflammatory biomarker in dogs, including both clinically healthy individuals and those with systemic inflammatory diseases. We aimed to evaluate the prognostic value of the ESR in dogs with inflammatory diseases and subsequently focused on dogs with SIRS to explore whether the ESR could serve as a rapid and practical prognostic tool for assessing mortality and disease severity in acute clinical settings.

## 2. Materials and Methods

### 2.1. Study Design and Population Selection

This study was designed as a retrospective observational cohort study. As this was an observational study, group allocation was based on clinical presentation and diagnosis, and thus randomization was not applied. The study was conducted in an unblinded manner, where clinicians and laboratory personnel were aware of the dog’s clinical status. The sample size was not predetermined by a statistical power analysis but was defined by the consecutive enrollment of all dogs that visited the hospital during the study period and met the specific inclusion and exclusion criteria detailed below. Dogs were included only when complete diagnostic information and full inflammatory marker panels were available, whereas cases were excluded if essential clinical data were missing or if concurrent diseases that could interfere with the interpretation of inflammatory markers were identified.

This study utilized blood samples collected from dogs that visited a single referral hospital between October 2023 and February 2025. All parameters were obtained at initial presentation. Cases were included only when the diagnostic assessment, as determined by the attending clinician, was sufficient to confirm a final diagnosis. In addition, dogs included in this study underwent comprehensive clinical evaluations including a physical examination and blood tests such as a complete blood count (CBC), serum biochemistry analysis, and blood gas analysis to confirm the absence of clinically relevant abnormalities To be included in the study, each dog was required to have concurrent measurements of all inflammatory markers—ESR, CRP, albumin-globulin ratio (A/G), neutrophil-lymphocyte ratio (NLR), and WBC count—as well as complete data for calculating the Acute Patient Physiologic and Laboratory Evaluation Fast (APPLE_fast_)score, including platelet count, plasma glucose, serum albumin, blood lactate concentration, and mentation score. The experimental design was approved by the University Institutional Animal Care and Use Committee (Approval number: CNU IACUC-YB-R-2025-42).

### 2.2. Animals

A population of 109 healthy dogs was included in this study (healthy group). These dogs were presented to a local animal hospital for routine health check-ups, neutering, dental scaling, or mild gastrointestinal symptoms such as transient anorexia. Owners provided written informed consent prior to participation, and blood sampling via jugular venipuncture was performed only after obtaining permission in accordance with institutional ethical guidelines. Healthy dogs were defined based on their clinical history, physical examination findings, CBC, and biochemical profile. A total of 241 dogs were classified as having underlying diseases (disease group). In these cases, the diagnosis was determined by the attending veterinarian based on their clinical history, physical examination findings, hematological and biochemical analyses, histopathologic examination, and imaging studies. These dogs presented with conditions requiring medical intervention including infectious diseases, endocrine disorders, cardiovascular diseases, and neoplastic conditions. For each case, the data collected included the breed, age, sex, neutering status, follow-up duration, and overall survival.

### 2.3. Criteria for Subgroup Categorization

A prognostic evaluation was conducted for subgroups within the disease group, specifically those including dogs with SIRS. SIRS subgroups were selected due to their strong pathophysiological association with systemic inflammation.

The dogs were classified into the SIRS group if they underwent a physical examination that included the measurement of rectal temperature, heart rate, respiratory rate, and WBC count with band neutrophil percentage. The SIRS group was defined by meeting at least two of the following criteria: rectal temperature (<38.1 °C or >39.2 °C), heart rate (>120 beats per minute), respiratory rate (>20 breaths per minute), and WBC count (<6 × 10^9^/L or >16 × 10^9^/L) with band neutrophils >3% [14].

### 2.4. ESR Measurement

The ESR assay was conducted using an automated ESR in the animal hospital (MINI-PET, DIESSE, Diagnostica Senese S.p.A., Monteriggioni, Italy) following the manufacturer’s guidelines. The analysis was performed within minutes after the same 1 mL K3-EDTA tube (Idexx Laboratories, Westbrook, ME, USA) had been used for a CBC evaluation (Idexx Procyte Dx, Idexx Laboratories, Westbrook, ME, USA) [15]. After the 14 min optical reading, the ESR result (mm/h) is displayed on the machine. The ESR was measured immediately following jugular venipuncture, and no storage of blood samples was performed prior to analysis.

### 2.5. Selection and Measurement of Inflammatory and Prognostic Biomarkers

In the selection of inflammatory biomarkers, four key indicators including CRP, NLR, A/G, and WBC count were chosen along with the ESR. These biomarkers were selected based on their established associations with systemic inflammation and their prognostic relevance, particularly in assessing disease severity and outcomes in various clinical conditions [1,2,4,7,9,10,11]. The values of these biomarkers for each case were determined based on the initial blood test results obtained at the time of the first presentation to the veterinary hospital, and the selected biomarkers were measured using the following methods. A CBC evaluation was performed using a 1 mL K3-EDTA tube (Idexx Laboratories, Westbrook, ME, USA) and analyzed with a hematology analyzer (Idexx ProCyte Dx, Idexx Laboratories, Westbrook, ME, USA). CRP, total protein, lactate, glucose and albumin were measured using an automatized biochemistry analyzer (Idexx Catalyst Dx, Idexx Laboratories, Westbrook, ME, USA) with plain serum tubes (Idexx Laboratories, Westbrook, ME, USA). The A/G was calculated as the albumin-globulin ratio, and the NLR was determined as the neutrophil-lymphocyte ratio.

In addition, for prognostic assessment, the APPLE_fast_ score was calculated for each patient based on five variables: platelet count, plasma glucose, serum albumin, blood lactate concentration, and mentation score, as previously described in the literature [16]. This composite scoring system has been validated as an early outcome predictor in critically ill dogs, integrating both hematobiochemical and clinical parameters.

### 2.6. Statistical Methods

Only dogs with complete measurements for the ESR, CRP, A/G, NLR, and WBC count were included. Similarly, dogs with missing data for any relevant biomarker measurements were excluded from the respective analyses including subgroup comparisons, correlation analyses, and prognostic assessments.

The normality of continuous variables, including the ESR, CRP level, NLR, and WBC count, was assessed using the Shapiro–Wilk test. Variables were classified as non-normally distributed if *p* < 0.05; otherwise, they were classified as normally distributed variables. Normally distributed variables were analyzed using an unpaired *t*-test, while non-normally distributed variables were analyzed using the Mann–Whitney U test. The comparison of the ESR, CRP level, NLR, and WBC count between the disease and healthy groups were evaluated using the unpaired *t*-test. Similarly, the ESR in the SIRS subgroup and healthy group was compared using the same method. Prognostic evaluation using the ESR in SIRS subgroups was conducted using the Mann–Whitney U test, as the data did not meet the conditions of normality.

To determine the optimal cut-off values for the ESR, ROC curve analysis was performed, and the corresponding sensitivities and specificities were calculated. The optimal cut-off values were determined using the Youden index (J), calculated as J = Sensitivity + Specificity − 1, to identify the threshold that maximizes the sum of sensitivity and specificity. In addition, ROC curve analysis was conducted for each biomarker to identify the threshold values that best discriminated between survivors and non-survivors.

The correlation between the ESR and inflammatory biomarkers (CRP level, A/G, NLR, WBC count) as well as hematocrit (HCT) was assessed using Pearson’s correlation analysis. Correlation analysis between age and ESR was also conducted in both the healthy and disease groups. We separately analyzed the correlation between ESR and age in the healthy and disease groups, as inflammatory status in diseased patients could confound this relationship. Although an age-related increase in ESR has been reported in humans [11], we aimed to clarify whether this association truly reflects an age effect or is instead influenced by the higher prevalence of illness in older individuals.

For Kaplan–Meier survival analysis, the entire study population was divided into quartiles based on the ESR. In addition, dogs in the SIRS subgroups were categorized into two groups (Group A: below the cut-off ESR value, Group B: equal to or above the cut-off ESR value) according to the optimal cut-off values derived from the ROC curve analyses. Statistical significance was assessed using the log-rank test.

To identify independent prognostic factors for all-cause mortality, Cox proportional hazards models were utilized. First, a univariate analysis was performed for each variable. Subsequently, variables were selected for the multivariate Cox regression model based on statistical significance in the univariate analysis (*p* < 0.1) or their established clinical relevance as key inflammatory or prognostic indicators (e.g., ESR, CRP, NLR, APPLE_fast_). This dual approach ensured that potential confounders were adjusted for, allowing for a robust evaluation of prognostic independence regardless of initial univariate thresholds. For this analysis, each dog was assigned a numerical value corresponding to the number of SIRS criteria met, with a score of 2, 3, or 4 given when two, three, or four criteria were satisfied, respectively. Signalment variables (age, sex, and body weight) were included in the multivariate models to adjust for their potential confounding effects.

All statistical analyses were conducted using two-tailed tests with a significance threshold set at 0.05. Statistical trends were defined for *p* values between 0.05 and 0.1. All statistical analyses and data processing were conducted using commercially available software, GraphPad Prism version 10.0 (GraphPad Software Inc., San Diego, CA, USA).

## 3. Results

### 3.1. Study Population

A total of 350 dogs including 109 healthy dogs (healthy group) and 241 dogs with various diseases (disease group) were enrolled in the study. The dogs in the healthy group were significantly younger (median age: 4.2 years; range: 0.2–15 year; IQR: 2–9.5 years) than the dogs in the disease group (median age: 10.1 years; range: 0.2–18 years; IQR: 6.3–12.5 years; *p* < 0.0001). In addition, the median hematocrit (HCT) was significantly lower in the disease group (median: 40.6%; IQR: 34.6–46.6%) compared to the healthy group (median: 46.6%; IQR: 41.5–51.3%; *p* < 0.0001). The participant demographics are detailed in Table 1. Of the 241 dogs in the disease group, 53 deceased and 188 survived. Dogs in the disease group that met the criteria for SIRS were selected for subgroup analyses, a cohort of 64 dogs diagnosed with SIRS was identified exclusively from within the disease group. This SIRS cohort was composed of 16 dogs that had died and 48 that had survived.

Among deceased dogs in the entire group, the median time to death (not median survival time) was 69 days (range:0–469, IQR: 21–146 days). Among the deceased dogs from the entire study population, excluding those with SIRS, the median time to death was 113 days (range: 1–305, IQR: 38–172 days). Among deceased dogs in SIRS subgroup, the median time to death was 52 days (range: 0–469, IQR: 14–143 days).

### 3.2. Comparison of Inflammatory Biomarkers Between the Healthy and Disease Groups

**Figure 1:**
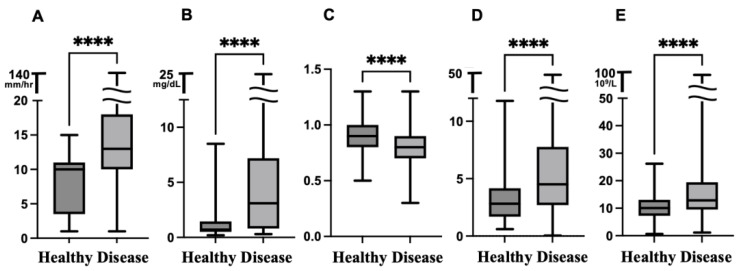
Comparison of inflammatory biomarkers between the healthy and disease groups. Box plots showing the distribution of inflammatory markers: (**A**) Erythrocyte sedimentation rate (mm/h), (**B**) C-reactive protein level (mg/dL), (**C**) Albumin-to-globulin ratio, (**D**) Neutrophil-to-lymphocyte ratio, (**E**) White blood cell count (10^9^/L). Note that panels C and D represent ratios and are therefore unitless. The horizontal line within each box represents the median value, and the top and bottom edges of the box indicate the interquartile range (IQR, 25–75th percentiles). The whiskers extend to the minimum and maximum values. The disease group showed significantly elevated levels of inflammatory markers compared to the healthy group (*p* < 0.0001). Statistical comparisons were performed using the unpaired *t*-test. Note the break in the y-axis for panels (**A**), (**B**), (**D**) and (**E**) to accommodate outliers. Significance levels are indicated as follows: **** *p* < 0.0001.

The median ESR was significantly higher in the disease group (median ESR: 13 mm/h; Interquartile range (IQR): 10–18 mm/h) than in the healthy group (median ESR: 10 mm/h; IQR: 4–11 mm/h; *p* < 0.0001). Similar significant differences were observed in other inflammatory markers including the CRP level, A/G, NLR, and WBC count between the disease and healthy group (all *p* < 0.0001, Table 2, Figure 1). ROC curve analysis comparing the ESR between the disease and healthy group yielded an AUC of 0.766 [95% CI:0.717–0.814], with an ESR cutoff value of 12 mm/h (sensitivity: 77.98%, specificity: 64.17%; *p* < 0.0001).

All inflammatory markers showed statistically significant differences between the disease and healthy groups (*p* < 0.0001), indicating that the disease group exhibited a markedly elevated inflammatory state compared with the healthy controls. The markers analyzed included: Erythrocyte sedimentation rate (ESR), C-reactive protein level (CRP), Albumin-to-globulin ratio (A/G), Neutrophil-to-lymphocyte ratio (NLR), and White blood cell (WBC) count. Statistical comparisons were performed using the unpaired *t*-test.

### 3.3. Correlation Analysis Between the ESR and Other Biomarkers

The results of the correlation analysis are reported in Table 3. The analyses indicated that the ESR was positively correlated with the CRP level, NLR, and WBC count, while it was negatively correlated with the A/G and HCT. All values showed a weak correlation (*p* = 0.006 to *p* <0.0001, *r* value: 0.1–0.3). The correlation between the ESR and other inflammatory biomarkers was consistent with previous reports (17). Although not statistically significant, Correlation analysis indicated a positive trend toward increasing ESR with advancing age in both the healthy and disease groups (0.05 ≤ *p* < 0.10).

### 3.4. ROC Curve Analysis

In the entire cohort, ESR demonstrated the strongest prognostic value as a single biomarker (ROC AUC = 0.776 [95% CI: 0.71–0.84]; Cut-off: 14 mm/h; Sensitivity: 74.41%, Specificity: 71.7%, *p* < 0.0001). Other inflammatory markers, including NLR, A/G ratio, WBC count, and CRP, were also significantly associated with survival (*p* < 0.05 for all). In contrast, the APPLE_fast_ score was not a significant predictor of mortality. The median values, *p* values, ROC AUCs, sensitivities, specificities, and cut-off values for each marker are detailed in Table 4.

The prognostic performance of the ESR was subsequently evaluated in the SIRS subgroup. For dogs diagnosed with SIRS, the ESR was significantly higher than that in healthy dogs (*p* < 0.0001) and showed the strongest prognostic value as a single biomarker within the SIRS group (ROC AUC = 0.846 [95% CI: 0.747–0.946]; Cut-off: 18 mm/h; Sensitivity: 77.08%, Specificity: 87.5%, *p* < 0.0001). The A/G ratio was also significantly associated with prognosis (ROC AUC = 0.727 [95% CI: 0.576–0.878]; Cut-off: 0.75; Sensitivity: 63.04%, Specificity: 75%, *p* = 0.005). Similarly, the APPLE_fast_ score demonstrated a statistically significant association with prognosis (ROC AUC = 0.672 [95% CI: 0.532–0.811]; Cut-off: 19 (score); Sensitivity: 41.67%; Specificity: 93.75%; *p* = 0.041), whereas other inflammatory markers including CRP, NLR, and WBC count were not significantly associated with survival. The median values, *p* values, ROC AUCs, sensitivities, specificities, and cut-off values for each inflammatory marker and prognostic indicator, including the APPLE_fast_ score, are detailed in Table 4.

### 3.5. Kaplan–Meier Survival Analyses

**Figure 2:**
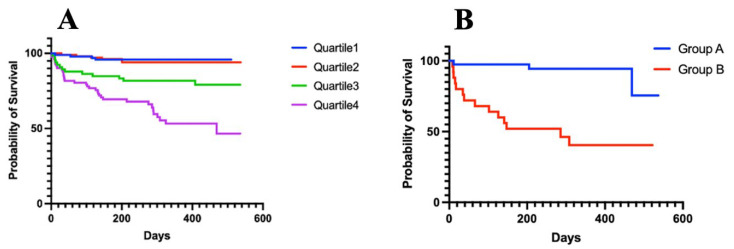
Kaplan–Meier survival analyses. Survival analyses were performed for the entire study population, stratified into quartiles (Q1–Q4) based on the ESR (**A**), with a maximum follow-up period of 537 days. For the erythrocyte sedimentation rate (ESR) analysis, cumulative survival rates progressively declined as the quartile increased (log-rank test for trend, *p* < 0.0001) (**A**). The median survival time (MST) was not reached (NR) for quartiles Q1–Q3, whereas the MST for Q4 was 469 days (95% Confidence Interval [CI]: 289—Not Estimated [NE]). (**B**) For the SIRS subgroup, dogs were categorized into two groups (Group A: below cut-off; Group B: ≥cut-off) using ROC-derived thresholds. In the ESR-based analysis of the SIRS group, the median survival time (MST) for Group A was not reached, while the MST for Group B was 286 days (95% CI: 66—Not Estimated [NE]). Log-rank tests showed significant survival differences between Group A and Group B in both the ESR analyses for the SIRS group (*p* < 0.0001).

Kaplan–Meier survival analyses were performed for the entire study population, stratified into quartiles (Q1–Q4) based on the ESR (Figure 2A) with a maximum follow-up period of 537 days. For both analyses, cumulative survival probabilities showed a clear and progressive decline as quartile scores increased, indicating poorer outcomes in dogs with higher ESR. In the ESR analysis, the log-rank test for trend confirmed a significant difference among quartiles (*p* < 0.0001), with the median survival time (MST) not reached (NR) for Q1–Q3 and 469 days (95% CI: 289–not estimated [NE]) for Q4.

Survival analyses were performed for the SIRS subgroup, stratified according to the optimal cut-off values derived from ROC analysis for ESR (18 mm/h) (Figure 2B). In the ESR-based analysis, dogs with ESR < 18 mm/h (Group A) had a median survival time (MST) that was not reached (NR), whereas those with ESR ≥ 18 mm/h (Group B) had an MST of 286 days (95% CI: 66–NE). Log-rank tests confirmed significant survival differences between Groups A and B in both analyses (*p* < 0.0001).

In the SIRS subgroup, elevated ESR were found to be independently and significantly associated with reduced survival probabilities.

### 3.6. Cox Proportional Hazards Analysis

The results of the Cox proportional hazards analyses are presented in Table 5.

In the univariate analysis for the entire group, age, ESR, CRP, A/G, NLR and the SIRS criteria, were significantly associated with mortality (*p* < 0.05), whereas the WBC count showed a statistical trend toward significance (0.05 ≤ *p* < 0.1). For the SIRS subgroup, univariate analysis identified age, ESR, A/G, APPLE_fast_ score as independent prognostic factors (*p* < 0.05; Table 5).

Subsequent multivariate analysis of the entire group revealed that age, ESR, NLR, and SIRS criteria were independent predictors of mortality. In the SIRS subgroup, age and the APPLE_fast_ score remained as independent prognostic indicators, while ESR showed a statistical trend toward significance (Table 5).

Notably, across all multivariate analyses, age consistently emerged as the most powerful independent prognostic predictor for mortality.

## 4. Discussion

This study evaluated the prognostic value of the ESR in dogs. Our study reconfirms ESR’s utility as an inflammatory marker and newly evaluates its prognostic role, aiming to establish the prognostic value of both ESR in systemic inflammatory conditions. Notably, only a limited number of contemporary veterinary studies have examined ESR dynamics in systemic inflammation or prognostic aspects [3,15,17]. Therefore, part of the mechanistic interpretation in the present study relies on established human literature, and some level of translational extrapolation remains unavoidable. Future work focusing specifically on canine haemorrheological responses and acute-phase protein interactions will be essential to strengthen species-specific evidence.

In veterinary medicine, the reference range for the ESR in healthy dogs is relatively low—generally reported between 1 and 10 mm/h [3,17]. In our study, the optimal cut-off value between the disease group and the healthy group was calculated as 12 mm/h; this result is similar to that from a previous report. The sensitivity was 77.98%, and the specificity was 64.17%, demonstrating a relatively low specificity consistent with previous reports [3] (Table 2).

The ESR has long been recognized as an inflammatory biomarker for disease diagnosis and monitoring [3]. It is widely employed in chronic inflammatory conditions such as rheumatoid arthritis and cancer [8,10]. The ESR reflects the overall burden of systemic inflammation rather than rapid acute-phase changes and thus is frequently used alongside acute-phase markers such as CRP and WBC counts, particularly in critical care settings like sepsis or SIRS. However, ESR is nonspecific, unable to distinguish inflammatory etiologies, and demonstrates a time-dependent limitation, rising 24–48 h after inflammation onset and remaining elevated for weeks despite resolution of the underlying cause. Rather than being solely a limitation, this characteristic allows the ESR to better indicate persistent or cumulative inflammation, complementing acute-phase indicators [3,17].

This cumulative reflection of inflammation by ESR is driven by slower-changing plasma proteins such as fibrinogen and immunoglobulins and influenced by plasma viscosity, erythrocyte aggregation (e.g., rouleaux, agglutination), and hematocrit levels [3,15]. In this study, the correlation between HCT and ESR was analyzed, confirming a statistically significant negative correlation (*r* = −0.306, *p* < 0.001). A reduced hematocrit can facilitate rouleaux formation and thereby accelerate the rate of erythrocyte sedimentation [15]. Accordingly, while the significantly lower HCT observed in the disease group may physiologically contribute to accelerated sedimentation, anemia should be viewed as an integral component of the systemic inflammatory process rather than a standalone confounder. Given the strong influence of fibrinogen established in previous studies, the observed ESR elevations likely reflect the cumulative inflammatory burden rather than hematocrit alterations alone [3,17].

Although ESR has been reported to increase with age in humans [3,11], this study did not reveal a statistically significant correlation between ESR and age in either the healthy or disease groups, despite a mild positive trend (0.05 ≤ *p* < 0.10). Further studies with longer follow-up periods may be needed to clarify this association (Table 3).

Correlations between ESR and other inflammatory markers vary among studies. Human medical research, which often considers specific disease conditions, infections, or timing factors, generally reports stronger correlations compared to veterinary studies [18,19,20]. Veterinary studies typically report weak to moderate correlations [3,17,21]. Our results align with previous veterinary studies reporting low correlation coefficients between ESR and CRP (previously r ≈ 0.18), although we observed a slightly higher correlation (r = 0.30) (Table 3). Similarly, statistically significant but weak correlations were observed between ESR and other biomarkers including CRP, NLR, WBC count, and A/G (*p* = 0.006 to *p* < 0.0001; *r* = 0.1–0.3), classified as weak (0.1–0.3), moderate (0.4–0.6), or strong (0.7–0.9) [17,22]. These weak correlations in veterinary medicine may be attributed to differing biomarker kinetics. Future studies should further explore temporal dynamics and disease-specific variations to improve the clinical interpretation of biomarkers.

In human medicine, recent studies have demonstrated that the ESR, along with other inflammatory markers, has significant potential as a prognostic indicator [10,11]. Large-scale cohort studies have shown that elevated ESR is independently associated with increased mortality irrespective of age [11]. Similar trends are observed in cancer patients, where elevated ESR correlates with poorer prognoses across various tumor types, including soft tissue sarcoma, renal cell carcinoma, and multiple myeloma [12,13,18,23]. Additionally, recent studies of critically ill patients admitted to intensive care units with severe infections—such as COVID-19—have highlighted ESR’s prognostic value, with significantly elevated ESR levels independently predicting disease severity and mortality [24]. These findings underscore the clinical utility of ESR in assessing disease progression and prognosis across diverse critical conditions.

In our prognostic analyses, the Kaplan–Meier survival analysis for the entire group clearly demonstrated that cumulative survival probabilities significantly decreased as the ESR quartiles increased, highlighting its role in long-term outcomes (Figure 2). Furthermore, ESR emerged as an independent and significant prognostic marker in this general cohort, as supported by both ROC curve analysis and multivariate Cox regression.

In our SIRS subgroup, ESR was significantly higher compared to the healthy group, indicating a markedly elevated inflammatory state in SIRS dogs (Table 2). ESR may not increase rapidly in acute systemic conditions such as SIRS [18]; however, recent evidence indicates its potential utility in acute settings, especially in patients with chronic comorbidities undergoing exacerbation [25,26]. This supports the notion that the ESR is not entirely insensitive in acute inflammatory states and can contribute prognostic value depending on the clinical context and underlying conditions. Dogs with SIRS are at risk of progressing to sepsis, making early identification and accurate risk assessment crucial [27,28]. In veterinary medicine, although ESR is not routinely measured in SIRS cases, our study demonstrated superior predictive performance for mortality compared to other inflammatory markers. In our study, ESR alone showed the highest prognostic accuracy in the SIRS subgroup ROC curve analysis, with a statistically significant association with mortality (*p* < 0.0001) (Table 4). In terms of prognostic capacity, ESR demonstrated superior performance compared to established markers in this cohort. While CRP and NLR are sensitive diagnostic markers, they did not show independent prognostic significance in our multivariate model. In contrast, ESR showed the highest AUC (0.846 [95% CI: 0.747–0.946]) in the SIRS subgroup, outperforming the A/G ratio (AUC 0.727 [95% CI: 0.576–0.878]). This suggests that while CRP reflects the immediate inflammatory status, ESR—driven by fibrinogen—better captures the cumulative severity that correlates with mortality [3,15,17]. Most importantly, this analysis provides a practical threshold for clinicians: an ESR cut-off of 18 mm/h predicted mortality with a high specificity of 87.5% in SIRS dogs. This high specificity is clinically significant, as it implies that a dog with SIRS presenting with an ESR above 18 mm/h has a strong likelihood of a poor outcome. This highlights the ESR’s value as a time-accumulated marker of systemic inflammation, particularly relevant in SIRS, where chronic and acute inflammation often coexist. These findings support the clinical utility of routine ESR measurement in dogs with SIRS, given its simplicity, low cost, and prognostic value in early risk stratification.

It is important to interpret the distinct ESR cut-off values identified in this study within their specific clinical contexts. The 12 mm/h threshold served as a diagnostic cut-off to differentiate diseased dogs from healthy individuals. In terms of prognosis, a cut-off of 14 mm/h was identified for predicting mortality in the overall disease cohort. Meanwhile, a higher threshold of 18 mm/h was determined for the SIRS subgroup, with high specificity (87.5%) to identify dogs at the greatest risk of death in acute conditions. Used alongside age and the APPLE_fast_ score, ESR adds a valuable layer of risk stratification.

Our Cox proportional hazards analysis identified several independent predictors of mortality. In the multivariate analysis of the entire cohort, age, ESR, NLR, and the presence of SIRS were identified as independent prognostic factors for survival, whereas the A/G ratio showed a trend toward significance (Table 5). This finding is consistent with previous reports identifying the presence of systemic inflammation as a prognostic factor influencing survival [7,8]. Of the single inflammatory markers evaluated, ESR was a key prognostic tool, proving to be a significant independent predictor in the entire group (*p* = 0.005). Ultimately, the evidence for ESR as a predictor of long-term mortality was most clearly established in the overall population analysis.

In the SIRS subgroup—a condition closely associated with poor outcomes—ESR also showed a trend toward significance (*p* = 0.062). This trend is particularly noteworthy as none of the other single inflammatory markers reached statistical significance in this severe cohort, suggesting ESR’s potential as a prognostic marker even in these critical cases. This trend is particularly noteworthy, as none of the other single inflammatory markers reached statistical significance within the SIRS subgroup. Although the statistical power of this subgroup analysis is limited by the modest sample size, ESR recorded the highest AUC (0.846 [95% CI: 0.747–0.946]) among single inflammatory biomarkers—outperforming CRP and NLR—and demonstrated the strongest prognostic association excluding the composite APPLE_fast_ score, thereby supporting its potential utility even in acute high-risk settings like SIRS.

The difference in prognostic performance of biomarkers between the overall and SIRS groups may be attributed to the pathophysiologic dynamics among biomarkers [19,20,25,26]. The APPLE_fast_ score, originally developed to predict short-term mortality [16], did not show strong prognostic performance in the entire cohort but demonstrated significant predictive power within the SIRS subgroup, which reflects an acute inflammatory state. This discrepancy may reflect differences in the predominant inflammatory status (chronic vs. acute) and survival timelines (long-term vs. short-term; specifically, the difference in median time to death of deceased patients between the entire group and the SIRS group) represented by the overall and SIRS groups, respectively.

In our study, age was consistently the most powerful independent predictor of mortality across all analysis. This finding aligns with numerous studies in veterinary medicine where advancing age is a key negative prognostic indicator across a wide range of conditions [29,30]. This is likely because advancing age likely reflects complex biological factors not fully captured by other biomarkers, such as a diminished physiological reserve to cope with severe illness, the gradual decline of the immune system (immunosenescence), and a higher likelihood of subclinical comorbidities [31,32]. Given this dominant influence, any biomarker like ESR that demonstrates significant prognostic value independent of age would be of considerable clinical importance.

Considering these results, the inclusion of ESR in future multi-biomarker prognostic scoring systems, analogous to the APPLE_fast_ score, could provide substantial clinical benefits. Given its demonstrated value, particularly for long-term prognosis in the general cohort, further research is warranted to investigate the development and validation of new, ESR-inclusive scoring models.

This study has several limitations. As a single-center study, our findings are potentially subject to selection bias and limited generalizability. The modest sample size, particularly within subgroups, may have restricted statistical power. Another limitation is the lack of covariate analysis for factors known to influence the ESR, such as serum protein status. Additionally, the Neutrophil–Lymphocyte Ratio (NLR) was calculated based on automated differential counts, and the lack of manual smear verification for all samples may have reduced the accuracy of this specific metric compared with manual counting. Moreover, the inherent non-specificity of the conventional SIRS diagnostic criteria raises the possibility that patients without true systemic inflammation were inadvertently included in the SIRS subgroup analysis, which may have affected the precision of the prognostic evaluation in this cohort. Furthermore, while the proposed cut-offs offer practical guidance for risk stratification, they should be interpreted with caution due to the retrospective nature of the study. In particular, the cut-off of 18 mm/h in SIRS patients prioritizes specificity, thereby identifying those at highest risk of mortality. It is also important to note that, although blood samples were collected at the initial hospital presentation before any in-hospital treatment commenced, we could not completely rule out the potential confounding influence of prior medication administration (e.g., corticosteroids or NSAIDs) or hydration status that may affect ESR [33]. Lastly, biological interpretations of ESR in this study partly rely on human literature due to the limited availability of veterinary ESR research, and thus some degree of translational extrapolation should be considered when interpreting these findings. Future studies should aim to validate the prognostic utility of ESR across diverse cohorts, especially in longitudinal designs assessing its trends over time. Such efforts would allow for deeper insights into treatment monitoring, relapse prediction, and long-term prognostication.

## 5. Conclusions

In conclusion, this study demonstrates an independent association between elevated ESR and mortality in the general disease cohort (*p* = 0.005). Our findings suggest a tiered clinical application: an ESR > 12 mm/h supports the diagnosis of systemic inflammation, while a cut-off of >14 mm/h indicates increased mortality risk in the general population. Notably, within the high-risk SIRS subgroup, an ESR cut-off of >18 mm/h exhibited superior prognostic performance compared to CRP and NLR, identifying patients at high risk of mortality with 87.5% specificity. These findings suggest that ESR serves as a practical, objective tool for risk stratification, reflecting the cumulative burden of systemic inflammation. Future prospective studies are warranted to validate these cut-offs across diverse disease etiologies.

## Figures and Tables

**Table 1:** Demographics of the study population of 350 dogs.

Category	Variables	Details
Age		Healthy group (*n* = 109); median 4.2 years; (range, 0.2–15 years, IQR: [2–9.5 years])
		Disease group (*n* = 241); median 10.1 years; (range, 0.2–15 years, IQR: [6.3–12.5 years])
Sex		Males (*n* = 178); *n* = 155 castrated, *n* = 23 intact
		Females (*n* = 172); *n* = 152 spayed, *n* = 20 intact
Breeds	Pure breeds (*n* = 298) and mixed breeds (*n* = 52)	Miniature Poodle (*n* = 56), Maltese (*n* = 55), Mixed (*n* = 52), Pomeranian (*n* = 46), Welsh Corgis and Bichon Frise (*n* = 24 each), Chihuahua (*n* = 16), Yorkshire Terrier (*n* = 12), Shih Tzu (*n* =9), Pug and Doberman Pinscher (*n* = 7 each), American Bully (*n* = 6), Old English Sheepdog (*n* = 5), Italian Greyhound, Golden Retriever, Coton de Tulear (*n* = 4 each), Spitz, French Bulldog, Cocker Spaniel (*n* = 3 each), Pekingese (*n* = 2), and Cane Corso, Brussels Griffon, Siberian Husky, Beagle, Shiba Inu, Miniature Pinscher, Labrador Retriever, Cavalier King Charles Spaniel (*n* = 1 each).
Underlying conditions	Disease group (*n* = 241)	Urogenital disease (*n* = 45): Chronic kidney disease (*n* = 42), Urinary calculus (*n* = 2), Cystitis (*n* = 1), Cardiovascular disease (*n* = 27): Myxomatous mitral valve disease (*n* = 27), Gastrointestinal disease (*n* = 24): Gastrointestinal foreign bodies (*n* = 7), Hemorrhagic gastroenteritis (*n* = 7), Gastric dilation and volvulus (*n* = 3), Pancreatitis (*n* = 3), Chronic enteropathy (*n* = 3), Triaditis (*n* = 1), Tumor (*n* = 21): Mammary gland tumor (*n* = 5), Mast cell tumor (*n* = 4), Splenic hemangiosarcoma (*n* = 3), Pheochromocytoma (*n* = 2), Pulmonary adenocarcinoma (*n* = 2), Squamous cell carcinoma (*n* = 1) Transitional cell carcinoma (*n* = 1), hemangiosarcoma (*n* = 1), Multicentric T-cell lymphoma (*n* = 1), Cutaneous lymphoma (*n* = 1), Hepatobiliary disease (*n* = 17): Chronic hepatitis (*n* = 10), Gallbladder rupture (*n* = 3), Hepatic encephalopathy (*n* = 2), Chronic pancreatitis (*n* = 1), Gallbladder mucocele (*n* = 1), Reproductive disease (*n* = 17): Pyometra (*n* = 15), Perianal hernia (*n* = 2), Musculoskeletal disease (*n* = 16): Medial patellar luxation (*n* = 7), Bone fracture (*n* = 3), Coxofemoral luxation (*n* = 3), Cruciate ligament (ACL) tear (*n* = 2), Degenerative arthritis (*n* = 1), Respiratory disease (*n* = 9): Pneumonia (*n* = 4), Aspiration pneumonia (*n* = 2), Respiratory distress (*n* = 2), Brachycephalic Obstructive Airway Syndrome (*n* = 1), Neurological disease (*n* = 8): Intervertebral Disc Disease (*n* = 6), Meningitis (*n* = 1), Idiopathic seizure (*n* = 1), Traumatic disease (*n* = 8): Fall trauma (*n* = 5), Bite wound (*n* = 2), Traffic accident (*n* = 1), Infectious disease (*n* = 7): Parvoviral enteritis (*n* = 5), Bacterial cystitis (*n* = 1), Clostridial enteritis (*n* = 1), Immune-mediated disease (*n* = 7): Immune mediated polyarthritis (*n* = 4), Immune mediated thrombocytopenia (*n* = 2), Systemic lupus erythematous (*n* = 1), Endocrine disease (*n* = 7): Hyperadrenocorticism (*n* = 5), Hypoadrenocorticism (*n* = 2), Hematologic disease (*n* = 3): Splenic torsion (*n* = 3), Ophthalmic disease (*n* = 3): Uveitis (*n* = 1), Corneal ulcer (*n* = 1), Glaucoma (*n* = 1), Dermatologic disease (*n* = 2): Atopic dermatitis (*n* = 1), Dermatophytosis (*n* = 1), Miscellaneous (*n* = 20): Post-surgical (*n* = 12), Pyrexia (*n* = 2), Onion toxicity (*n* = 2), Abdominal mass (*n* = 1), Splenic mass (*n* = 1), Unclassified multiple dermatologic mass (*n* = 1), Unclassified multiple abdominal mass (*n* = 1)

**Table 2:** Inflammatory marker comparison in the disease and healthy groups.

Characteristic		ESR (mm/h)	CRP (mg/dL)	A/G	NLR	WBC Count (10^9^/L)
Median Value (IQR)	Healthy group (*n* = 109)	10 (4–11)	0.7 (0.5–1.4)	0.9 (0.8–1.0)	2.82 (1.69–4.07)	10.07 (7.29–12.99)
	Disease group (*n* = 241)	13 (10–18)	3.1 (0.8–7.2)	0.8 (0.7–0.9)	4.52 (2.70–7.74)	12.93 (9.54–19.49)
	SIRS Subgroup (*n* = 64) ^1^	15 (12–31)	7 (3.35–9.1)	0.8 (0.7–0.9)	5.305 (2.82–8.525)	15.55 (10.78–22.13)
Diagnostic performance (Healthy vs. Disease group)						
ROC AUC (95% CI) *		0.766 (0.717–0.814)	0.769 (0.707–0.832)	0.661 (0.597–0.724)	0.715 (0.656–0.773)	0.680 (0.619–0.740)
Specificity (%) *		77.98	75.41	36.45	71.28	70.21
Sensitivity (%) *		64.17	65.89	88.24	61.16	58.93
Cut-off value *		12	1.5	0.9	3.715	11.43
*p* value *		<0.0001	<0.0001	<0.0001	<0.0001	<0.0001

^1^ SIRS subgroup is a subset of the disease group and is shown for descriptive purposes only. * *p* values, ROC AUC, specificity, sensitivity, and cut-off values represent the statistical comparison between the healthy group and the disease group.

**Table 3:** Pearson’s correlation between the erythrocyte sedimentation rate (ESR) and other investigated inflammatory markers.

	CRP	A/G	NLR	WBC count	HCT	Age (Healthy)	Age (Disease)
Correlation coefficient (*r*)	0.302	−0.235	0.206	0.154	−0.306	0.160	0.117
*p* value	<0.0001	<0.001	<0.001	0.006	<0.0001	0.097	0.070

C-reactive protein level (CRP), albumin-to-globulin ratio (A/G), neutrophil-to-lymphocyte ratio (NLR), and white blood cell (WBC) count, and hematocrit (HCT). All correlations were statistically significant (*p* = 0.006 to *p* < 0.0001), with correlation coefficients (r) ranging from 0.1 to 0.3.

**Table 4:** Comparison of Prognostic Accuracy of Inflammatory Markers and Acute Patient Physiologic and Laboratory Evaluation Fast (APPLE_fast_) Score.

		ESR (mm/h)	CRP (mg/dL)	A/G	NLR	WBC Count (10^9^/L)	APPLE_fast_ Score (Points)
Entire group							
Median Value (IQR)	Alive (*n* = 53) Deceased (*n* = 297)	11 (8–14) 16 (13–36)	1.6 (0.6–5.4) 3.8 (1.2–6.8)	0.8 (0.7–0.9) 0.7 (0.6–0.8)	3.45 (2.24–5.96) 7.00 (4.31–11.21)	11.19 (8.65–15.05) 14.92 (9.51–23.3)	18 (17–20) 19 (16–21)
*p* value		<0.0001	0.013	<0.0001	<0.0001	0.002	0.684 (ns)
ROC AUC (95% CI)		0.776 (0.709–0.843)	0.612 (0.535–0.689)	0.703 (0.629–0.776)	0.740 (0.667–0.814)	0.638 (0.550–0.726)	NE
Sensitivity (%)		74.41	54.02	68.59	67.79	87.64	NE
Specificity (%)		71.7	66.67	60.78	70.59	40.38	NE
Cut-off value		14	2.05	0.7	4.82	20.23	NE
SIRS subgroup							
Median Value (IQR)	Alive (*n* = 48) Deceased (*n* = 16)	14 (11–17) 34 (20–52)	7.1 (3.4–9.1) 6.7 (3.6–8.2)	0.8 (0.7–0.9) 0.7 (0.5–0.8)	5.34 (2.68–8.5) 5.19 (3.95–8.49)	15.7 (10.97–21.02) 15.2 (10.75–22.98)	18.5 (15–21) 22 (17–24)
*p* value		<0.0001	0.645 (ns)	0.005	0.526 (ns)	0.959 (ns)	0.041
ROC AUC (95% CI)		0.846 (0.747–0.946)	NE	0.727 (0.576–0.878)	NE	NE	0.672 (0.532–0.811)
Sensitivity (%)		77.08	NE	63.04	NE	NE	41.67
Specificity (%)		87.5	NE	75	NE	NE	93.75
Cut-off value		18	NE	0.75	NE	NE	19

The markers analyzed included the ESR, C-reactive protein level (CRP), albumin-to-globulin ratio (A/G), neutrophil-to-lymphocyte ratio (NLR), and white blood cell (WBC) count. In both the entire cohort and the systemic inflammation response syndrome (SIRS) subgroup, the erythrocyte sedimentation rate (ESR) demonstrated the strongest prognostic performance among single inflammatory biomarkers (*p* < 0.0001). In contrast, the APPLE_fast_ score failed to demonstrate significant prognostic value in the entire cohort, whereas it showed statistical significance in the SIRS subgroup. ns = No significance. NE = Not evaluated.

**Table 5:** Cox proportional hazards analysis identifying predictors of all-cause mortality among signalment, inflammatory, and prognostic markers. Data are presented as hazard ratio (HR) and 95% confidence interval (CI). ns = No significance.

Entire group (*n* = 350)								
Univariate ^1^	HR	95% CI	*p* value	Multivariate ^2^	HR	95% CI	*p* value	
Age (year)	1.197	1.128–1.276	<0.0001	Age (year)	1.163	1.088–1.247	<0.0001	
Sex	0.848	0.527–1.357	0.491 (ns)	Sex	1.604	0.9223–2.820	0.944 (ns)	
Body weight (kg)	1.013	0.940–1.079	0.712 (ns)	Body weight (kg)	1.002	0.916–1.082	0.963 (ns)	
ESR	1.024	1.017–1.030	<0.0001	ESR	1.013	1.004–1.022	0.005	
CRP	1.104	1.030–1.181	0.006	CRP	0.992	0.897–1.092	0.865 (ns)	
A/G	0.044	0.011–0.184	<0.0001	A/G	0.211	0.037–1.208	0.080	
NLR	1.153	1.098–1.205	<0.0001	NLR	1.084	1.109–1.150	0.012	
WBC count	1.014	0.999–1.026	0.060	WBC count	1.001	0.976–1.022	0.929 (ns)	
SIRS Criteria	1.551	1.348–1.788	<0.0001	SIRS Criteria	1.275	1.033–1.576	0.024	
APPLE_fast_ score	1.006	0.919–1.095	0.889 (ns)	APPLE_fast_ score	1.043	0.931–1.156	0.452 (ns)	
SIRS subgroup (*n* = 64)								
Univariate ^1^	HR	95% CI	*p* value	Multivariate ^2^	HR	95% CI	*p* value	
Age (year)	1.274	1.108–1.501	<0.001	Age (year)	1.198	1.013–1.461	0.035	
Sex	1.943	0.712–5.306	0.190 (ns)	Sex	0.948	0.254–3.535	0.936 (ns)	
Body weight (kg)	1.095	0.921–1.275	0.288 (ns)	Body weight (kg)	1.071	0.861–1.285	0.510 (ns)	
ESR	1.030	1.016–1.043	<0.001	ESR	1.018	0.999–1.036	0.062	
CRP	1.053	0.889–1.140	0.552 (ns)	CRP	1.098	0.889–1.350	0.389 (ns)	
A/G	0.015	0.001–0.302	0.006	A/G	0.733	0.023–27.120	0.862 (ns)	
NLR	1.017	0.893–1.140	0.789 (ns)	NLR	1.004	0.862–1.151	0.959 (ns)	
WBC count	0.994	0.950–1.020	0.734 (ns)	WBC count	0.990	0.931–1.031	0.656 (ns)	
APPLE_fast_ score	1.157	1.013–1.334	0.031	APPLE_fast_ score	1.251	1.034–1.546	0.020	

Abbreviations: A/G, albumin-globulin ratio; CI, confidence interval; CRP, C-reactive protein; ESR, erythrocyte sedimentation rate; HR, hazard ratio; NLR, neutrophil-lymphocyte ratio; SIRS, systemic inflammatory response syndrome; WBC, white blood cell. ^1^ Univariate analysis. ^2^ Multivariate analysis including all variables shown.

## Data Availability

The original contributions presented in this study are included in the article.

## Abbreviations

APPLE_fast_	Acute Patient Physiologic and Laboratory Evaluation Fast
A/G	Albumin-Globulin (ratio)
AUC	Area Under the Curve
CBC	Complete Blood Count
CI	Confidence Interval
CRP	C-Reactive Protein
ESR	Erythrocyte Sedimentation Rate
HCT	Hematocrit
HR	Hazard Ratio
IACUC	Institutional Animal Care and Use Committee
IQR	Interquartile Range
K3-EDTA	Tripotassium Ethylenediaminetetraacetic Acid
NE	Not Estimated
NLR	Neutrophil-Lymphocyte Ratio
ns/NS	No significance/non-significant
MST	Median Survival Time
RBC(s)	Red Blood Cell(s)
ROC	Receiver Operating Characteristic (curve)
SIRS	Systemic Inflammatory Response Syndrome
WBC	White Blood Cell (count)
